# Nitrogen Fertilizer Deep Placement for Increased Grain Yield and Nitrogen Recovery Efficiency in Rice Grown in Subtropical China

**DOI:** 10.3389/fpls.2017.01227

**Published:** 2017-07-11

**Authors:** Meng Wu, Guilong Li, Weitao Li, Jia Liu, Ming Liu, Chunyu Jiang, Zhongpei Li

**Affiliations:** ^1^State Key Laboratory of Soil and Sustainable Agriculture, Institute of Soil Science, Chinese Academy of Sciences Nanjing, China; ^2^Graduate University of Chinese Academy of Sciences Beijing, China

**Keywords:** N deep placement, N broadcast, grain yield, N recovery efficiency, ^15^N tracing

## Abstract

Field plot experiments were conducted over 3 years (from April 2014 to November 2016) in a double-rice (*Oryza sativa L.*) cropping system in subtropical China to evaluate the effects of N fertilizer placement on grain yield and N recovery efficiency (NRE). Different N application methods included: no N application (CK); N broadcast application (NBP); N and NPK deep placement (NDP and NPKDP, respectively). Results showed that grain yield and apparent NRE significantly increased for NDP and NPKDP as compared to NBP. The main reason was that N deep placement (NDP) increased the number of productive panicle per m^-2^. To further evaluate the increase, a pot experiment was conducted to understand the N supply in different soil layers in NDP during the whole rice growing stage and a ^15^N tracing technique was used in a field experiment to investigate the fate of urea-^15^N in the rice–soil system during rice growth and at maturity. The pot experiment indicated that NDP could maintain a higher N supply in deep soil layers than N broadcast for 52 days during rice growth. The ^15^N tracing study showed that NDP could maintain much higher fertilizer N in the 5–20 cm soil layer during rice growth and could induce plant to absorb more N from fertilizer and soil than NBP, which led to higher NRE. One important finding was that NDP and NPKDP significantly increased fertilizer NRE but did not lead to N declined in soil compared to NBP. Compared to NPK, NPKDP induced rice plants to absorb more fertilizer N rather than soil N.

## Introduction

To obtain high yields of cereal crops, high rates of N fertilizer are applied in China (Liang et al., 2013). However, farmers usually apply excessive N fertilizer that is not proportional to crop yield (Peng et al., 2006; Shen et al., 2013). This practice causes ammonia volatilization, denitrification, surface runoff, and leaching in paddy cultivation systems leading to low N recovery efficiency (NRE) (De Datta and Buresh, 1989; Wang et al., 2001) and has also become a threat to the environment in China (Shi et al., 2012). Improving NRE is one of the main objectives in modern agriculture (Yousaf et al., 2016).

Many experiments have provided evidence that deep placement of N fertilizer could effectively increase grain yield and NRE (Kapoor et al., 2008; Bandaogo et al., 2015) as compared to N broadcast application (NBP) in lowland rice fields. The main topics of these studies have been: (1) determining that N fertilizer deep placement could reduce ammonia volatilization compared to broadcast application of N fertilizer (Rochette et al., 2013) and thus increase NRE (Huda et al., 2016); (2) seeking appropriate N application materials such as urea supergranules and briquette N (Mohanty et al., 1999; Gaudin and D’Onofrio, 2015) for N fertilizer deep placement; (3) identifying other specific agronomic practices for N fertilizer deep placement such as optimizing the depth of N fertilizer placement in different countries (Mohanty et al., 1999; Gaudin, 2012); (4) and developing and applying fertilization machines for deep fertilizing (Bautista et al., 2001; Liu et al., 2015). Few of these studies focus on the N distribution during rice growth under N fertilizer deep placement. However, the distribution of N with deep fertilizer placement is probably the main reason for higher grain yield and NRE compared to NBP.

In China, broadcast application of fertilizers is still the common practice for lowland rice. Recently, the deep placement method of N fertilizer has attracted much attention in China (Xu et al., 2013; Kargbo et al., 2016). In the present study, a pot experiment was carried out to investigate the N supply in different soil layers during the whole growth duration of rice under N deep placement (NDP) and NBP. Three-year field plot experiments were carried out (from 2014 to 2016) including NBP, NDP, and N, P, K deep placement (NPKDP) under double-rice cropping systems in the middle and lower reaches of the Yangtze river in subtropical China, and the grain yield and NRE were determined. The ^15^N tracer technique was used to investigate the N distribution during rice growth and to determine the N fate of urea-^15^N in the rice–soil system as influenced by fertilization method during early rice growing in the field experiments in 2016.

## Materials and Methods

### Pot Experiment for NDP

A pot experiment was conducted in a greenhouse (32°03′32″N, 118°48′18″E) at Nanjing, Jiangsu Province (Southern China) using soil taken from the field described in the following section. The soil was air-dried, pulverized, and mixed before filling the pots. Each pot (a plastic box with length 30 cm × width 20 cm × height 30 cm) contained 15 kg of soil and a hill of rice. The rice was transplanted in late July and three seedlings (4 weeks old) were used per hill. Three treatments, CK (no N fertilizer), NBP (broadcast application of N fertilizer), and NDP (NDP at 12 cm right below soil surface where the rice seedling was placed) were conducted with three replicates. In each pot, the base fertilizer, urea (N 0.92 g per pot for N treatments), calcium magnesium phosphate (P_2_O_5_ 0.50 g per pot), and potassium chloride (K_2_O 0.83 g per pot) were applied according to planting density and the rate of fertilizer application in the field experiment. For CK and NBP, the fertilizers were broadcasted on the soil surface. For NDP, P, and K were broadcasted and N fertilizer was deep applied. First, a hole was dug with a depth of 12 cm in each pot. The N fertilizer was point-placed at 12 cm depth and a tubule with many small holes and 300 mesh nylon fabric wrapped around it was placed in a circle around where the N fertilizer was applied. Then the soil was filled into the hole and another tubule was placed at 5 cm depth just above the N fertilizer locus. After fertilization, the pots were all flooded and the rice seedlings were transplanted; the duration between this operation and harvest was 120 days. The soil solution at 5 cm and 12 cm depths were drawn with a syringe through the two tubules. At day 3, 7, 11, 17, 23, 37, 52, 67, and 107, the surface water and soil solution at 5 cm and 12 cm depths were sampled and measured for NH_4_^+^-N concentration with a continuous flow analyzer (Skalar, Netherlands).

### Description of Field Experimental Area

**FIGURE 1:**
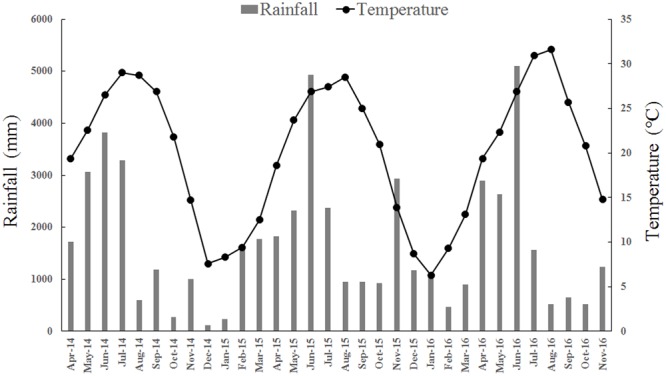
Monthly total rainfall and monthly mean temperature during the rice growing season in the experiment conducted during 2014–2016.

The field plot experiment was conducted in Sixi town, Jiangxi province (115°09′32″E, 28°32′29″N). Annual average temperature is 17.6°C, annual precipitation is 1700 mm, and the frost-free season is 276 days. The meteorological data during the rice-growing period are shown in **Figure 1**. Soil in the test field is paddy soil derived from river alluvial deposits, classified as Fe-accumul-Stagnic Anthrosols in Chinese Taxonomy. Soil samples were collected just before early rice in 2014, and the soil properties at 0–20 cm depth were: soil organic carbon (SOC) 20.5 g kg^-1^; total nitrogen (N) 1.75 g kg^-1^ (a high N level); total phosphorus (P) 0.65 g kg^-1^; total potassium (K) 27.7 g kg^-1^; available N 191 mg kg^-1^; available P 42.6 mg kg^-1^; available K 92.0 mg kg^-1^; pH 4.96; and bulk density 1.32 g cm^-3^. Early rice (conventional indica rice, zhongjiazao17) was transplanted in late April and harvested in late July. Late rice (conventional rice indica, wufengyouT025) was transplanted immediately after early rice and was harvested in November of 2014, 2015, and 2016 in the present study.

### Field Experimental Design and Operation

We used four treatments based on a completely randomized design with three replicates in field experiments: (1) no N fertilizer, as a control (CK); (2) conventional broadcast of N fertilizer (NBP); (3) N and (4) NPK deep placement at 12 cm below the soil surface (NDP and NPKDP, respectively), Length × width of each plot was 6 × 7 m in treatments CK and NBP and 2 × 2 m in treatments NDP and NPKDP. Planting density in each plot was equivalent to 1.8 × 10^5^ basic seedling ha^-1^. The N fertilizer used was commercial granular urea and was applied at a rate of 135 kg ha^-1^ (amounting to 1.6 g urea per hill) and 165 kg ha^-1^ (amount to 2.0 g urea per hill) for early and late rice, respectively. Split N fertilization was performed in NBP with 40% basal, 30% tiller, and 30% boot N input for early and late rice, while urea was used as the single basal N fertilizer in NDP and NPKDP treatments. Calcium-magnesium phosphate (P_2_O_5_ 90 kg ha^-1^) and potassium chloride (K_2_O 150 kg ha^-1^) were amended as single basal fertilizers for all treatments. Water in the plots of NDP and NPKDP treatments was drained as much as possible, and rice seedlings were transplanted immediately. Then, urea was placed 12 cm below the soil surface where rice seedlings were placed by self-owned stainless fertilization cylinder. Finally, the plot was flooded again and P and K fertilizers were broadcast to the flooded soil surface.

### Sampling and Measurements for Field Experiment

Just before the grain harvest, three hills of plants were collected in each plot to determinate the stem length, productive panicle number per m^2^ (PPN) and spikelet number per panicle (SNP). Then the sampled plants were first dried at 105°C for 30 min and then dried at 75°C until a constant weight was achieved. The thousand grain weight was recorded, and the straw and grain were ground through a 0.5-mm sieve to test their N concentration. Grain yield and straw biomass in each plot were weighted during the harvest, and then the water content of grain and straw were also tested.

### ^15^N Tracing Experiment in the Field

The ^15^N tracing experiment was conducted during the early rice season in 2016. Two microplots (about 0.5 m × 0.5 m), bounded and separated by plastic frames in each plot, were fertilized with urea labeled with 10.22 atom % excess ^15^N provided by the Shanghai Research Institute of Chemical Industry. The amount and application method of ^15^N urea was the same as the operation in the field experiment described above. The plastic frames were pressed to the bottom of the plow layer and kept about 8 cm above the soil to reduce lateral flow of water and ^15^N. Four hills of rice seedlings were transplanted in each microplot to monitor the fates of ^15^N-labeled fertilizer. At 35 days after transplanting, the aboveground parts of the plant as well as the soil at depths of 0–5 cm, 5–15 cm, and 15–20 cm under each rice plant in one microplot from each plot were collected. Samples in the same soil layer of the same microplot were mixed and treated as one replicate. At rice maturity, rice plants in the other microplot in each plot were harvested by hand. The aboveground parts of plants were separated into grain and straw. Roots in each microplot were all collected from the 0–20 cm soil layer and washed with water. In the meantime, soil at depths of 0–20 cm was also collected in each microplot. All plant samples were dried at 75°C to constant weight to determinate their dry weight, and the soil samples were air-dried, then ground to powder and passed through a 150-μm screen for N content and ^15^N analysis. The grain, straw, root, and soil samples were analyzed for total N and ^15^N abundance using an elemental analyzer (Vario MAX, Elementar Analysensysteme GmbH, Germany) and an isotope-ratio mass spectrometer (Delta V Advantage, Thermo Fisher Scientific Inc., United States).

### Calculation and Interpretation of ^15^N Data

The ^15^N was expressed as the atom percent excess corrected for background abundance (0.366%). Plant N derived from fertilizer (Ndff) and Urea-N recovery (UNR) were calculated with the following formulas (López-Bellido et al., 2005; Chen et al., 2016):

(1) $$\begin{array}{c} \mathrm{Ndff}\%=(B-A)/(C-D)\times 100\;\; \end{array}$$

(2) $$\begin{array}{c} \mathrm{Ndff}({\mathrm{kgha}}^{-1},\mathrm{for}\mathrm{grain},\mathrm{straw},\mathrm{root}\mathrm{or}\mathrm{soil})=\mathrm{Ndff}\%\times N_{t} \end{array}$$

(3) $$\begin{array}{c} \mathrm{UNR}\%={Ndff/N}_{f}\times 100\left(3\right)\; \end{array}$$

where A is the atom% ^15^N in the unlabeled grain, straw, root or soil; B is the ^15^N atom% excess in the labeled grain, straw, root or soil; C is the ^15^N atom percent excess in the ^15^N fertilizer; D is natural ^15^N atom percent in the N fertilizer; N_t_ (kg ha^-1^) is the total N in the grain, straw, root or soil at maturity; and N_f_ is the amount of fertilizer N applied.

Apparent NRE and the ^15^NRE, were calculated as following formulas (Wang et al., 2011; Ruisi et al., 2015):

(4) $$\begin{array}{c} \mathrm{NRE}(\%)=(\mathrm{plant}\;N\;\mathrm{uptake}\;\mathrm{in}\;N\;\mathrm{treatment}\;(\mathrm{kg}\;{\mathrm{ha}}^{-1})\;-\mathrm{plant}\;N\;\mathrm{uptake}\;\mathrm{in}\;N0\;\mathrm{control}\;(\mathrm{kg}\;{\mathrm{ha}}^{-1}))/\mathrm{applied}\;N\;(\mathrm{kg}\;{\mathrm{ha}}^{-1})\times 100\; \end{array}$$

(5) $$\begin{array}{c} {}^{15}NRE(\%)\;=\frac{{\mathrm{plant}}^{15}N\;\mathrm{uptake}\;(\mathrm{kg}\;{\mathrm{ha}}^{-1})}{{\mathrm{applied}}^{15}N\;(\mathrm{kg}\;{\mathrm{ha}}^{-1})\times 100}\;\; \end{array}$$

### Data Analysis

Mean grain yield, yield composition, N use efficiency and soil nutrient were analyzed by one way analysis of variance (ANOVA). Stepwise regression analysis was applied to examine the yield composition that explained the highest variance in grain yield. Statistical analyses were carried out using SPSS 19.0, and significance (*P* < 0.05) was determined with the Duncan method.

## Results

### N Supply under Deep Placement in Pot Experiment

**FIGURE 2:**
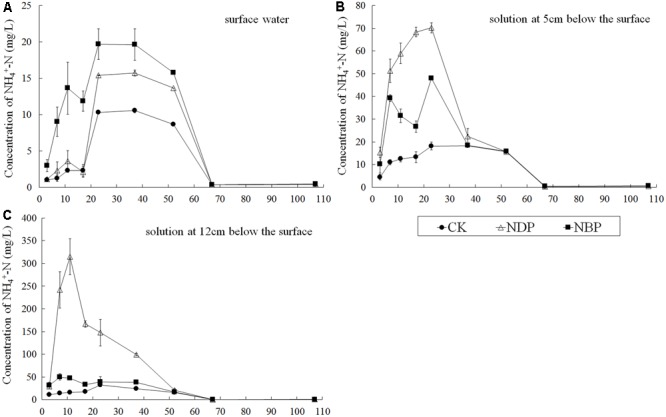
Comparison of NH_4_^+^-N concentration of the surface water **(A)**, the soil solution at 5 cm **(B)** and 12 cm **(C)** below the surface between N broadcast and N deep-point application at day 3, 7, 11, 17, 23, 37, 52, 67, and 107 in the pot experiment. CK, no N fertilizer as a control; NBP, conventionally applied broadcast N fertilizer; NDP, N deep application 12 cm below soil surface where the rice seedling was placed.

The differences in N supply between NDP and NBP were evaluated over the whole rice growth duration in the pot experiment. **Figure 2** shows that NH_4_^+^-N concentrations of NBP and NDP decreased to the low level of the control (CK) after 52 days in all three soil layers. NBP had a significantly higher NH_4_^+^-N concentration than NDP in the surface water, while NDP displayed a much higher NH_4_^+^-N concentration at depths of 5 cm (15.3–70.2 mg L^-1^) and 12 cm (20.8–314.8 mg L^-1^) than NBP (10.2–48.0 mg L^-1^ at 5 cm and 17.0–50.2 mg L^-1^ at 12 cm depth) during the first 52 days. The NH_4_^+^-N concentration of NDP followed the order 12 cm depth > 5 cm depth > surface water with significant difference. From day 7 to 37, the NH_4_^+^-N concentration of NDP ranged from 99.3 to 314.8 mg L^-1^ at 12 cm depth and from 15.7 to 70.2 mg L^-1^ at 5 cm depth, which also indicated that the N concentration sharply decreased at 7 cm away from the fertilizer location.

### Effect of N Fertilizer Placement Methods on Grain Yield and NUE

The average early and late grain yields of NDP and NPKDP in 3 years were about 8.41–10.10 t ha^-1^ higher than NBP, a significant (*P* < 0.05) increase of 35.7–53.5% (**Table 1**). NBP and CK exhibited similar grain N contents that were significantly lower (*P* < 0.05) than NDP and NPKDP. There were no statistically significant differences (*P* > 0.05) in straw biomasses and straw N content between NDP, NPKDP, and NBP for early and late rice. For the yield components among the treatments, CK had the smallest productive PPN and the lowest SNP. NDP and NPKDP showed significantly higher PPN (275.2–362.5 per m^2^) than NBP (185.6–256.5 per m^2^) with increases of 35.1–58.4%. There were no significant differences in SNP between NDP, NPKDP, and NBP, and stem length and thousand-grain weight (TGW) were not significantly different among the four treatments. NDP and NPKDP demonstrated much higher NRE (58.7–84.6%) than NBP with increases ranging from 116.9 to 194.8%.

**Table 1:** The average grain yield, yield composition and nitrogen use efficiency under different fertilization strategies in 3 years.

Treatment	Grain yield (t ha^-1^)	Straw biomass (t ha^-1^)	Grain N content (g kg^-1^)	Straw N content (g kg^-1^)	PPN (m^-2^)	SNP	TGW (g)	Stem length (cm)	NRE (%)
Early Rice									
CK	3.67 d	2.35 b	10.7 b	7.8 a	149.5 c	127.3 b	23.0 a	89.8 a	—
NBP	6.13 c	3.51 a	11.1 b	9.9 a	185.6 b	149.4 a	23.1 a	94.0 a	32.0 b
NDP	8.41 b	3.91 a	13.7 a	9.0 a	275.2 a	152.5 a	22.8 a	93.5 a	69.4 a
NPKDP	9.40 a	4.06 a	14.1 a	9.1 a	294.0 a	165.6 a	22.6 a	97.1 a	84.6 a
Late Rice									
CK	4.72 c	3.00 b	10.8 b	6.8 b	186.0 c	112.6 b	23.4 a	96.3 a	—
NBP	6.74 b	3.86 ab	11.6 b	8.3 ab	256.5 b	136.5 a	23.4 a	97.7 a	21.2 b
NDP	9.14 a	4.19 a	13.3 a	11.3 a	346.3 a	127.9 a	23.7 a	99.6 a	58.7 a
NPKDP	10.10 a	4.38 a	13.4 a	9.4 ab	362.5 a	134.6 a	23.5 a	99.3 a	62.5 a

*PPN, productive panicles numbers per m^-2^; SNP, spikelet numbers per panicle; TGW, thousand grain weight (g); NRE, apparent recovery efficiency of N (%). Within a column for each year, means followed by different letters are significantly different at the 0.05 probability level according to Duncan’s multiple comparison test.*

A stepwise regression analysis was conducted in order to identify the variable that explained most of the variance in grain yield in the regression model. The grain yield was considered the dependent variable and the PPN, SNP, TGW, and stem length were treated as independent variables. The results showed that PPN was the variable explaining the highest variance in grain yield (*y* = 0.027 × PPN + 0.387, *R*^2^ = 0.842, *P* < 0.001). A stepwise regression analysis was conducted by taking NRE as the dependent variable and grain yield, straw biomass, grain N, and straw N content as independent variables. The results showed that grain yield was the most important variable to explain the variance in NRE (%) for both early (*y* = 0.016 × Grain yield – 65.6, *R*^2^ = 0.913, *P* < 0.001) and late rice (*y* = 0.013 × Grain yield – 65.3, *R*^2^ = 0.955, *P* < 0.001).

### Plant N Uptake and N Distribution in Soil after the Tillering Stage

The plants and soil samples were collected at 35 days after rice transplanting when the tillering stage ended. NDP and NPKDP significantly increased the total N content and ^15^N abundance in plants compared to NBP (**Table 2**). Thus, the aboveground parts of plants from treatments NDP and NPKDP accumulated much more N than NBP. In the accumulated N, the plant N derived from soil (Ndfs) was not significantly different between NDP and NBP. However, the plant Ndff in NDP and NPKDP were significantly higher than in NBP, and the UNR also showed the same tendency.

**Table 2:** The nitrogen source of aboveground parts of plants in different treatments at 35 days after rice transplanting.

Treatment	TN (mg kg^-^)	^15^N (%)	Nt (kg ha^-^)	Ndff (%)	Ndff (kg ha^-^)	Ndfs (kg ha^-^)	UNR
NBP	19.3 b	3.1 b	44.6 b	29.8 b	13.3 b	30.0 a	14.1 b
NDP	27.3 a	6.0 a	77.3 a	56.8 a	43.8 a	33.5 a	32.5 a
NPKDP	25.3 a	6.6 a	81.0 a	62.9 a	51.0 a	31.3 a	37.7 a

*N_t_ (kg ha^-1^) means the total N in the plant; Ndff and Ndfs mean the plant N derived from fertilizer and soil; UNR means the urea-N recovery efficiency. Different letters means significant difference at the 0.05 probability level according to Duncan’s multiple comparison test.*

In addition, the soil N content was not significantly different at 0–5 cm and 5–15 cm depths between NBP, NDP, and NPKDP (**Table 3**). However, the NPKDP treatment had significantly higher N content at 15–20 cm than NDP and NBP. At the 0–5 cm soil layer, the Ndff of NBP was the highest and the Ndff of NPKDP was much lower than that of NBP and NDP. At the 5–15 and 15–20 cm soil layers, the Ndff of NBP was very low and the Ndff of NPKDP was higher than that of NDP. Generally, most of the Ndff (84.7%) was retained in the 0–5 cm soil layer in the NBP treatment. For NDP, Ndff was the highest in the 5–15 cm soil layer (37.5%). NPKDP, on the other hand, retained the most Ndff (42.2%) at the 15–20 cm soil layer.

**Table 3:** The nitrogen distribution in different soil layers in different treatments at 35 days after rice transplanting.

Treatment	Soil (0–5 cm)				Soil (5–15 cm)				Soil (15–20 cm)			
	TN (g kg^-^)	Ndff (%)	Ndff (kg ha^-^)	FNP (%)	TN (g kg^-^)	Ndff (%)	Ndff (kg ha^-^)	FNP (%)	TN (g kg^-^)	Ndff (%)	Ndff (kg ha^-^)	FNP (%)
NBP	2.1 a	1.7 a	23.3 a	84.7 a	1.7 a	0.2 b	3.9 b	13.3 b	1.5 b	0.1 b	0.6 b	2.0 c
NDP	2.6 a	1.2 ab	20.5 a	37.3 b	1.9 a	0.9 a	22.0 a	37.5 a	1.5 b	1.4 a	14.3 a	25.2 b
NPKDP	2.1 a	0.9 b	12.4 b	18.4 c	1.8 a	1.1 a	27.5 a	39.4 a	2.0 a	2.0 a	26.6 a	42.2 a

*Ndff means the soil N derived from fertilizer; ^15^N (mg kg^-1^) = TN (mg kg^-1^) ×^15^N (%); FNP (%) means the amount of fertilizer-N in one soil layer accounts for the proportion of the total amount of fertilizer-N in the three soil layers, for example, FNP_0-5_ (%) = Ndff_0-5_/(Ndff_0-5_ + Ndff_5-15_ + Ndff_15-20_) × 100. Different letters means significant difference at the 0.05 probability level according to Duncan’s multiple comparison test.*

### Fate of Urea-^15^N in the Plant-Soil System at Rice Maturity

Fertilization practice significantly affected fertilizer N accumulation in rice. NPKDP showed significantly higher Ndff in grain, straw, and root than NBP and NDP, and NBP displayed much lower Ndff in grain, straw, and root than NDP (**Table 4**). The grain Ndfs of NBP was significantly lower than NDP and NPKDP, which meant that more N from soil went into the rice grain in the NDP treatments. However, the straw Ndfs of NBP was higher than NDP and NPKDP, which meant that more N from soil went into rice straw in the broadcast treatment.

**Table 4:** The nitrogen source of rice plants and soil residual urea-^15^N in different treatments at maturity.

Treatment	Grain			Straw			Root			Soil (0–20 cm)		N decline (kg ha^-^)
	Ndff (%)	Ndff (kg ha^-^)	Ndfs (kg ha^-^)	Ndff (%)	Ndff (kg ha^-^)	Ndfs (kg ha^-^)	Ndff (%)	Ndff (kg ha^-^)	Ndfs (kg ha^-^)	Ndff (kg ha^-^)	Ndff (%)	
NBP	20.3 c	12.9 c	51.0 c	13.4 c	4.3 c	30.5 a	8.6 a	0.23 b	2.9 a	19.2 b	0.43 a	59.2 a
NDP	33.7 b	39.2 b	77.2 a	32.6 b	11.0 b	22.8 a	9.9 a	0.74 ab	6.2 a	30.6 a	0.60 a	62.5 a
NPKDP	46.5 a	59.0 a	68.0 b	38.9 a	14.1 a	22.2 a	10.7 a	0.82 a	7.9 a	21.4 ab	0.49 a	60.1 a

*Ndff and Ndfs mean the N derived from fertilizer and soil; N decline (kg ha^-1^) is the soil N declined as a result of plant uptake. N decline = Ndfs in grain + Ndfs in straw – (Ndff + Ndfs) in root – Ndff in soil. Different letters means significant difference at the 0.05 probability level according to Duncan’s multiple comparison test.*

Previous work had indicated that most soil Ndff (>90%) was located in the 0–20 cm soil layer in paddy fields (Liu et al., 2016). Thus, in the present study we only evaluated the soil N in the 0–20 cm soil layer. NBP contained lower Ndff in soil than NDP and NPKDP and that meant that NDP treatments were preferred to retain more fertilizer N in soil than NBP. The N decline was calculated as the difference in plant N taken away from soil and fertilizer N retained in the soil (including N absorbed by roots). The N decline ranged from 59.2 to 62.5 kg ha^-1^ and was not significantly different among NBP, NDP, and NPKDP.

The total urea-^15^N recovery (U^15^NR) of rice plants and soil (0–20 cm) in NPKDP was 70.7%, which was significantly higher than NDP (60.4%) and NBP (27.4%). The U^15^NR in straw and root showed no significant difference between broadcast and NDP. The main difference was that the grain U^15^NR was highest in NPKDP and lowest in NBP. NBP also had the lowest soil U^15^NR and NDP had the highest soil U^15^NR. The NRE of NBP was 38.7%, which was significantly lower than NDP (76.9%), while the NRE of NPKDP was up to 86.5% and was significantly higher than NDP. However, the ^15^NRE calculated by the labeled method, which represented plant uptake of N directly from fertilizer, was much lower than NRE. The results indicated that the real NRE of NBP was only 12.8% and could increase to 37.2–54.2% through N or NPKDP.

## Discussion

### Grain Yield and N Use Efficiency

The average grain yield and NRE over 3 years indicated that fertilizer NDP could significantly improve the grain yield and NRE as compared to NBP. Similar effects of fertilizer placement on grain yield were also observed by Sano et al. (2008) in a paddy field in northeastern Japan. For broadcast application, it is well known that N, P, K balanced fertilization is more beneficial for maintaining high grain yield than N, P, or K deficient fertilization (Ji et al., 2015). Similarly, our result indicated that N, P, K balanced deep placement (NPKDP) could also further increase grain yield and NRE compared to NDP. By investigating yield compositions, we found that the main difference between NDP, NPKDP, and NBP lay in the number of productive panicle per m^2^ (PPN). The stepwise regression analysis showed that PPN accounted for the variation of grain yield, and grain yield mainly determined the differences in apparent NRE between different treatments. NDP improved PPN because it increased effective tiller numbers at earlier growth stages compared to NBP (Savant and Stangel, 1998). To further explore the reasons for differences in PPN, a pot experiment was conducted to investigate N supply in NDP during the entire rice growing season. In addition, the plant N uptake and N distribution in different soil layers just after the tillering stage in the field experiment were further investigated using the ^15^N tracing technique.

### N Distribution in Plants and Soil

According to the pot experiment, NDP at 12 cm below the soil surface could maintain high NH_4_^+^-N concentration at 5 cm and 12 cm depth in the soil solution for 52 days (at the end of the jointing stage). Liu et al. (2016) also determined that NDP could maintain higher soil NH_4_^+^-N concentration in different soil layers at 30 and 60 days after transplanting compared to NBP. As shown in **Figure 2**, the NH_4_^+^-N concentration remained at a low level and gradually increased during the first 7 days after transplanting. Since the seedling root was about 5 cm long and the mineral N concentration was relatively low at 5 cm depth in the beginning, seedling burning could be avoided. In this early vegetative stage, rice plants need relatively low levels of nutrients and should be allocated less N (Peng et al., 2006). After 7 days, as the NH_4_^+^-N concentration increased, the fertilizer N diffusion could meet the demand of rice growth in later stages. Thus, NDP could provide fertilizer N more efficiently and could improve grain yield and NRE. Our previous research indicated that N could migrate about 7 cm in about 35 days after applying N fertilizer on the soil surface (Li et al., 2013). In this study, the NH_4_^+^-N concentrations in soil solution at 5 cm depth sharply decreased compared to 12 cm depth where the fertilizer was located in 7–52 day, which indicated that N could also migrate about 7 cm in deep placement.

In the field experiment, a big difference in rice N uptake occurred at the end of the tillering stage. NDP and NPKDP absorbed much more fertilizer N than NBP, which indicated that NDP was more beneficial for rice to absorb fertilizer N during the vegetative growth stage. As the N absorbed by rice plants during the vegetative growth stage could contribute to the reproductive and grain filling stages via N translocation (Fageria and Baligar, 2005), NDP and NPKDP had more potential for high yield than NBP. In addition, the fertilizer N was mainly retained at the surface soil layer (0–5 cm) for NBP. For NDP and NPKDP, more fertilizer N was retained at deeper soil layers (5–20 cm) and this was quite beneficial for rice plants to absorb N when roots were proliferating in the later growing stages. Jing et al. (2012) indicated that high nutrient concentrations in the crop root-zone could increase root proliferation and thus increase crop yield. Adequate N supply from the jointing to booting stage plays a key role in increasing rice yield. From the N distribution in soil after the tillering stage, it was found that NDP could increase the N content in deeper soil layers (5–20 cm) and thus could increase grain yield compared to NBP. NPKDP could further increase plant N uptake and retain more N in the 15–20 cm soil layer than NDP, and thus has more potential for higher rice yield and N use efficiency than NDP.

Compared to NBP, the rice plants were more efficient at uptake of both fertilizer N and soil N under NDP at maturity, and this led to higher rice yield and N use efficiency. The grain and straw Ndff of NPKDP were significantly higher than in NDP, while the grain Ndfs of NPKDP was significantly lower than in NDP. That meant that NPKDP caused rice plants to absorb fertilizer N more efficiently and also reduced the N uptake from the soil compared to NDP. The results of urea-^15^N recovery also indicated that NDP caused a higher proportion of fertilizer N to accumulate in grains than NBP, especially NPKDP, which caused grains to absorb up to 43.7% of the applied fertilizer N. NDP significantly enhanced rice NRE according to different calculation methods (2.0–2.2 times higher on average of NRE and 2.9–4.2 times higher on average of ^15^NRE) compared to NBP (**Table 5**). Previous research also found that NRE could be improved through altering fertilizer placement (Sano et al., 2008). The traditional NRE value was higher than ^15^NRE from the ^15^N labeled method, which is most likely the result of positive priming and substitution effects between fertilizer N and soil N caused by immobilization (Zhao et al., 2009). In addition, NRE is usually susceptible to control (CK) plot effects. Thus, it is clear that using ^15^NRE to reappraise fertilizer N in-season use efficiency is more accurate than NRE.

**Table 5:** Effects of N fertilization practice on urea-^15^N recovery in rice plants and ^15^N use efficiency at maturity.

Treatment	Urea-^15^N recovery (%)				^15^NRE (%)	NRE (%)
	Grain	Straw	Root	Soil (0–20 cm)	Total		
NBP	9.6 b	3.2 b	0.17 a	14.2 b	27.2 c	12.8 c	38.7 c
NDP	29.0 ab	8.2 ab	0.55 a	22.7 a	60.4 b	37.2 b	76.9 b
NPKDP	43.7 a	10.5 a	0.61 a	15.9 ab	70.7 a	54.2 a	86.5 a

*Different letters means significant difference at the 0.05 probability level according to Duncan’s multiple comparison test.*

Previous research has demonstrated that soil total N declined after consecutive cropping for some years of using inorganic fertilizer (Xue et al., 2014). In this study, we found that rice grain and straw absorb more N from soil than fertilizer N reserved into soil, which would cause soil N declining. While the soil N declined in NBP, NDP and NPKDP were in the same level, which indicated that NDP and NPKDP only increased fertilizer NRE but did not induce N to decline compared to NBP.

## Conclusion

A 3-year, consecutive field plot experiment indicated that NDP increased early and late rice yield and NRE significantly compared to the NBP method in subtropical China. The stepwise regression analysis showed that PPN explained most of the variation of grain yield, which further determined the difference of NRE between different treatments. Pot experiments and a field ^15^N tracing study indicated that NDP could maintain high fertilizer N supply in deep soil layers (5–20 cm) and induced rice plants to absorb more N and produce more PPN than NBP. NDP induced rice to absorb more N from fertilizer and soil and also reserved more fertilizer N in soil than NBP. NDP and NPKDP increased fertilizer NRE but would not cause N to decline compared to NBP. Our results also showed that NPKDP utilized fertilizer N more efficiently, and exhibited higher grain yield than NDP. So N, P, K balanced deep placement is an attractive management practice for double-rice cropping in this region.

## Author Contributions

MW and ZL initiated and designed the research. MW, GL, WL, and JL performed the experiments and collected the data, MW analyzed the data and wrote the manuscript. ML and CJ edited the manuscript and provided guidance during experimentation.

## Conflict of Interest Statement

The authors declare that the research was conducted in the absence of any commercial or financial relationships that could be construed as a potential conflict of interest.
